# Safety of palbociclib concurrent with palliative pelvic radiotherapy: discussion of a case of increased toxicity and brief review of literature

**DOI:** 10.1002/jmrs.435

**Published:** 2020-09-28

**Authors:** Archya Dasgupta, Arjun Sahgal, Ellen Warner, Gregory J. Czarnota

**Affiliations:** ^1^ Department of Radiation Oncology Sunnybrook Health Sciences Centre Toronto Ontario Canada; ^2^ Department of Radiation Oncology University of Toronto Toronto Ontario Canada; ^3^ Physical Sciences Sunnybrook Research Institute Toronto Ontario Canada; ^4^ Department of Medical Oncology Sunnybrook Health Sciences Centre Toronto Ontario Canada; ^5^ Department of Medicine University of Medicine Toronto Ontario Canada; ^6^ Department of Medical Biophysics University of Toronto Toronto Ontario Canada

**Keywords:** CDK4/6 inhibitor, gastrointestinal toxicity, palbociclib, palliative radiotherapy, pelvic radiotherapy

## Abstract

Several cyclin‐dependent kinase 4/6 (CDK4/6) inhibitors are indicated in the treatment of metastatic hormone receptor‐positive (HR)/ human epidermal growth factor receptor 2 (HER2) negative breast cancer which includes palbociclib, ribociclib and abemaciclib. Pelvic radiation therapy (RT) is often indicated for symptomatic or progressive bone metastasis. There are limited data on concurrent use of CDK4/6 inhibitors with pelvic RT with few retrospective studies in the literature involving a small number of patients. The major side effects of these agents include haematological toxicities, while non‐haematological toxicities are less severe. There are concerns for an increased possibility of synergistic toxicity with concurrent use of CDK4/6 inhibitors with pelvic RT. Here we describe an instance of acute grade 3 gastrointestinal toxicity and discuss the relevant literature. A 77‐year‐old lady treated with palliative conventional RT 30 Gy/ 10 fractions concurrently with palbociclib to left hemipelvis and proximal femur, developed severe pancolitis starting 5 days from last RT. She needed inpatient care for 3 weeks and recovered with mesalamine and supportive care. We also postulate a few strategies that can be adopted in patients receiving palliative RT in such a scenario. The agents should be stopped 1 week before, during and for a time (1 week minimally) after RT. A shorter course of 5 fractions (and ablative RT as indicated) can be considered to minimise treatment gaps. Highly conformal techniques (intensity‐modulated radiotherapy/ volumetric‐modulated arc therapy) can significantly reduce bowel dose and should be considered in patients with pre‐existing GI comorbidities or prior GI toxicity with these agents.

## Graphical abstract

Cyclin‐dependant kinase 4/6 (CDK 4/6) inhibitors used concurrently with pelvic radiotherapy in metastatic breast cancer can lead to increased gastrointestinal toxicity. Careful approach is required with stopping of the drugs 1 week before, during and after the radiation; and conformal techniques can be considered to reduce bowel dose in patients with a higher risk of adverse effects.

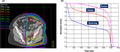

## Introduction

Palbociclib, taken in conjunction with an antiestrogen agent, is indicated for the treatment of hormone receptor‐positive (HR+)/ human epidermal growth factor receptor 2 negative (HER2–) metastatic breast cancer and was approved by the Food and Drug Administration (FDA) on an accelerated basis in 2015.^1^, ^2^, ^3^ Palbociclib is a selective inhibitor of cyclin‐dependent kinases 4/6 (CDK4/6) controlling the G1/S checkpoint of the cell cycle. In patients with HR + breast cancer, oestrogen signalling works coupled with cyclin D‐CDK4/6‐INK4‐Rb pathways.^4^ Thus the use of CDK4/6 inhibitors along with endocrinal therapy, can lead to more efficient blockade of cell division and overcome resistance to hormonal therapy alone. The standard regimen is oral administration once daily for 3 weeks, followed by 1 week off, in a 28‐day cycle. The most common grade 3 or 4 adverse event when used along with letrozole^2^ or fulvestrant^1^ is neutropenia (50% to 65%), although febrile neutropenia is rare. Minor toxicities include fatigue, nausea, arthralgias and anaemia. Although median progression‐free survival (PFS) in the 1^st^ line setting is approximately 24 months, almost double the PFS with hormonal therapy alone, drug resistance and progressive disease eventually occur.^2^, ^5^

Radiation therapy (RT) plays an integral role in the palliation of metastatic breast cancer, and there is emerging evidence of a positive impact of ablative RT on survival for patients with oligometastatic disease.^6^, ^7^ Bones, particularly the spine and pelvis, are the most frequent site of metastatic disease in HR + HER2‐ breast cancer. Patients with newly diagnosed metastatic disease are frequently started on systemic therapy with palbociclib plus either an aromatase inhibitor or fulvestrant, and concurrently referred for palliative RT to symptomatic sites of disease. It is also not uncommon for patients who have had a fairly long duration of disease response or stability from endocrine therapy plus palbociclib, but develop progressive disease at one or two bone sites, to be referred for palliative RT to that site while the systemic therapy is left unchanged with the goal of delaying the switch to chemotherapy. With pelvic bones and sacrum accounting for approximately 35% active bone marrow in adults^8^ and the radiation field to these areas including a significant amount of bowel,^9^, ^10^, ^11^ there may be concerns of synergistic haematological as well gastrointestinal (GI) toxicity when RT is delivered concurrently with palbociclib. Being a relatively newer drug introduced in clinical practice within the last 5 years, we have limited data regarding the safety of combining RT with palbociclib.^12^, ^13^, ^14^, ^15^, ^16^, ^17^ We present an instance of accelerated GI toxicity in a patient receiving palliative RT to pelvis concurrently with palbociclib and letrozole and subsequently discuss the related literature. Consent for the use of de‐identified patient information was obtained as per institutional policy (Sunnybrook Health Sciences Centre).

## Case Discussion

**Figure 1:**
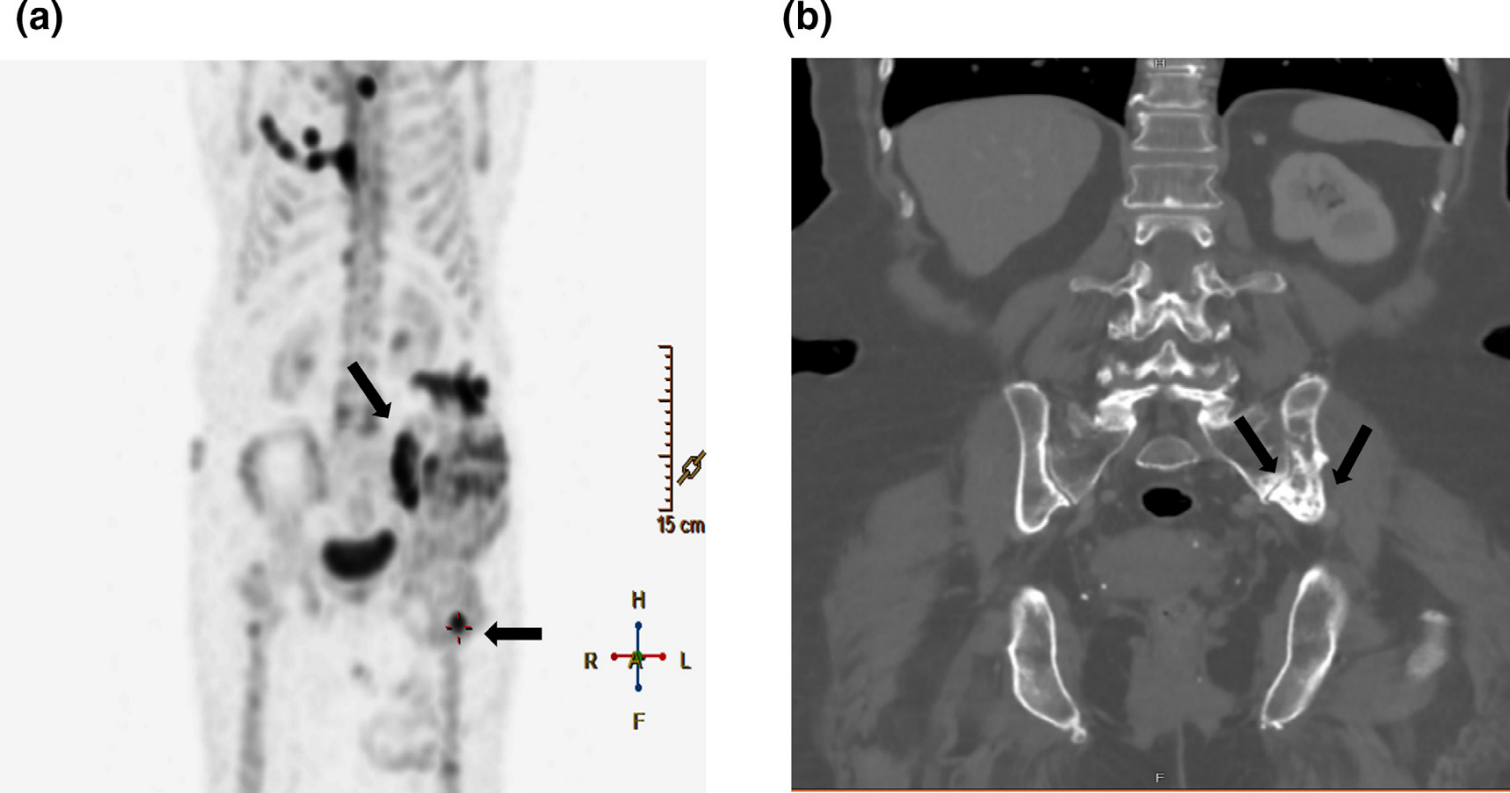
Bone scintigraphy (A) and computed tomography coronal view (B) showing the site of metastatic disease in bone involving left ilium and left femur (represented with arrows).

A 77‐year‐old lady was diagnosed initially with left‐sided HR+/HER2‐ breast cancer, for which she had undergone mastectomy and axillary lymph node dissection revealing a 3.5 cm invasive ductal carcinoma, grade 2, with six lymph nodes involved with macrometastasis out of 10 dissected. She was treated with adjuvant chemotherapy (5‐fluorouracil, epirubicin, cyclophosphamide and docetaxel). She received adjuvant radiotherapy to the left chest wall and ipsilateral regional nodal volumes (axilla, supraclavicular fossa, internal mammary chain) 50 Gy in 25 fractions according to standard institutional practice. She continued endocrine therapy with exemestane for 6 years until she had a recurrence involving the subcutaneous left chest wall and multiple bones (spine, pelvis) associated with mild intermittent pain. She was switched to tamoxifen, which resulted in good pain relief and disease control. After 3 years, she developed clinically asymptomatic progression of disease in her bones and chest wall. She was started on palbociclib (100 mg orally, 3 weeks on, 1 week off) and letrozole (2.5 mg daily). She tolerated the regimen well without major toxicities (mild arthralgia during the initial few months) and did not require any dose modification. After 30 months, she complained of increasing left hip pain. The bone scan was unchanged from previous imaging, but computed tomography (CT) of chest, abdomen, pelvis demonstrated oligoprogression with an increase in size of a prior metastasis in the left ilium, and a new area involving the left femoral head (Fig. 1). There was no evidence of visceral metastases. She was referred to the radiation oncology clinic. She described mechanical pain of moderate‐intensity related to the iliac metastasis that was not relieved by regular use of analgesics. She denied any limitation in her activities of daily living. Her comorbidities included hypertension, hyperlipidaemia and type 2 diabetes mellitus (well controlled on oral medications).

**Figure 2:**
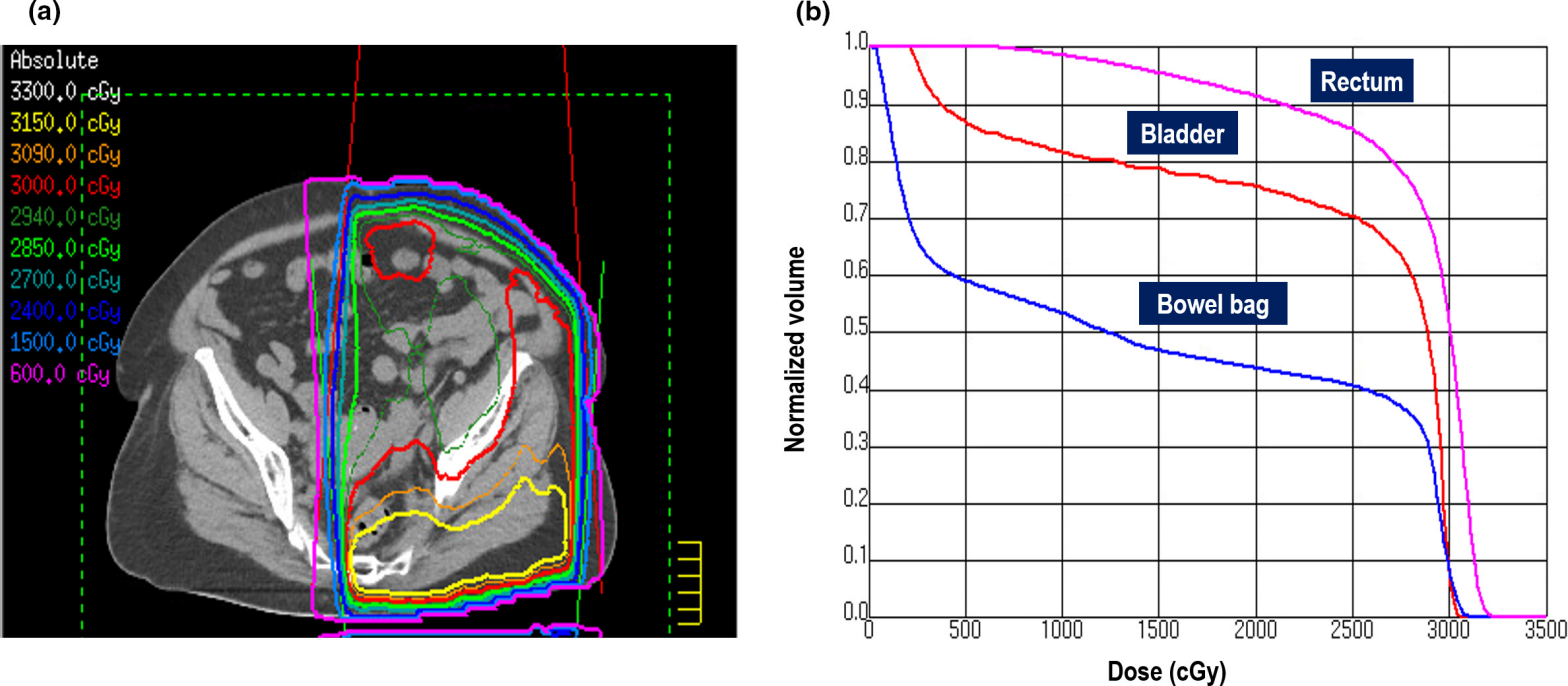
The isodose curves for the radiation plan on the planning computed tomography axial view (A). The dose volume histograms for bowel bag, rectum and urinary bladder are shown in Figure B.

**Figure 3:**
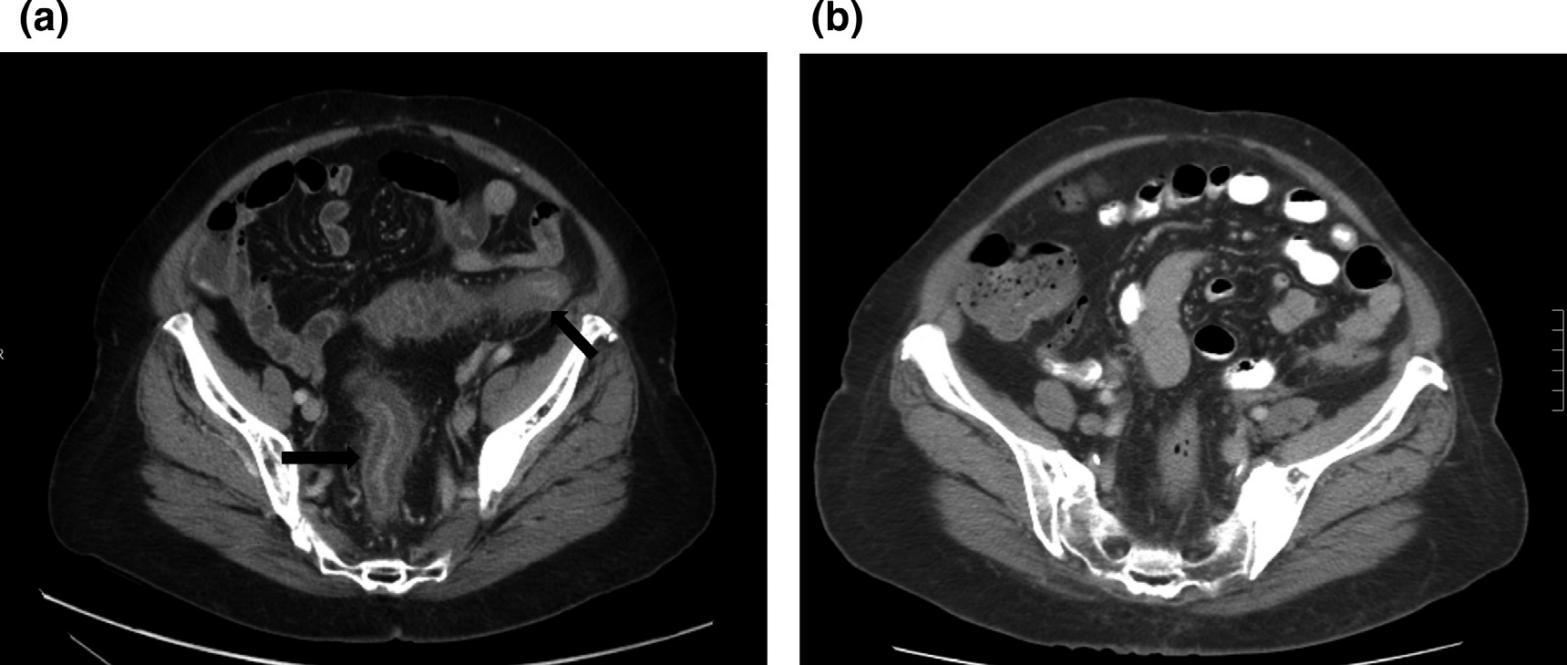
Figure A is the computed tomography axial view done at 5 days after completion of radiation (during admission) showing colitis represented as thickening of mucosa most apparent in the descending colon and rectosigmoid junction (arrows). Figure B shows the repeat computed tomography at 3 weeks from radiation completion with a resolution of the mucosal thickening.

She was presented with the options of either stereotactic body radiotherapy (SBRT) or conventionally fractionated radiotherapy (CRT), and the pros and cons of both techniques were discussed with her. She preferred CRT and received 30 Gy in 10 fractions over 2 weeks using parallel opposed portals to the left hemipelvis and proximal femur. The maximum dose delivered to bowel bag and rectum was 30.98 Gy and 32.18 Gy, respectively (RT isodose and dose volume histogram shown in Fig. 2). It was decided to include intervening areas of disease involving the left pelvis along with the target areas of progressive disease. She continued her palbociclib‐letrozole during RT, as she had tolerated it very well previously. Five days following completion of RT, she presented to the emergency with acute onset bloody diarrhoea and severe abdominal cramping without any fever. She was managed in the inpatient setting, and investigations turned out negative for any infective aetiology. CT abdomen showed generalised thickening of the colon predominantly affecting the rectum, sigmoid and descending colon suggestive of pancolitis (Fig. 3). Colonoscopy demonstrated fragile mucosa extending 20 cm from the anal verge, and biopsy showed inflammatory changes. The patient was treated with mesalamine in tapering dose (10 mg daily for 1 week, 2.5 mg daily 1 week) and supportive care. Her symptoms had sufficiently improved 3 weeks after admission to enable discharge and she reported significant improvement of her left hip pain. Palbociclib was held during the entire course of inpatient care and for the subsequent 3 weeks. Letrozole was switched to exemestane. Last follow‐up (4 months post‐RT), she remained free of gastrointestinal symptoms but complained of recurrence of pain in her left hip. Exemestane will be discontinued and replaced with fulvestrant.

## Discussion

We have recently witnessed a paradigm shift in the systemic therapy of cancer with the introduction of newer targeted agents, immunotherapies, cell cycle inhibitors and antiangiogenic therapies. The biological behaviour and clinical consequences of these agents, when used along with RT, need to be better understood, as there are concerns of possible increased toxicity from interactions.^18^, ^19^ The CDK4/6 inhibitors currently approved for use in HR + HER2‐ metastatic breast cancer include palbociclib, ribociclib and abemaciclib, and their clinical indications are likely to expand in the coming years.^20^ Patients with HR + metastatic breast cancer usually have protracted survival, particularly when the disease is confined to bone only, in which case the median survival can extend to five years or longer.^21^, ^22^ Therefore, a significant proportion of patients may require RT to the bony pelvis while they are on treatment with CDK4/6 inhibitors. It is crucial to establish safe and effective protocols for combining RT with new targeted drugs, to avoid escalating toxicities and minimise interruption of systemic therapy.

There are few reports available related to the use of CDK4/6 inhibitors concurrent with palliative RT, and those reports involve a limited number of patients. Chowdhury et al. described 16 patients receiving RT to various metastatic sites within 2 weeks of use of palbociclib.^15^ The most common indication was bone metastases in 11 patients (including 4 pelvic RT), brain metastasis in 4 patients and mediastinal RT in 1 patient. The authors reported excellent tolerance with only grade 2 haematological toxicities in 2 patients, all other toxicities (cutaneous, neurologic and GI) were grade 1 only. Another series by Ippolito et al. reported the use of palbociclib and ribociclib concurrently with RT in 13 and 3 patients (total 24 courses of treatment), respectively, including RT to the pelvis in 6.^16^ Grade 3 or higher toxicity was haematological (neutropenia) in 5 patients (sites treated‐humerus, lumbar spine, chest wall, femur neck, scapula), with 60% of them experiencing neutropenia during the previous cycles of palbociclib/ ribociclib. No GI toxicity was observed in any patients from 6 courses of treatment to pelvis and 4 to the abdomen (spine). In a recent series by Beddok et al., palbociclib was used in 30 patients, and the most common toxicities reported were radiation dermatitis and neutropenia.^17^ Nine patients received palbociclib concurrently with loco‐regional RT and palbociclib was discontinued in two patients due to toxicity (grade 3 dermatitis and febrile neutropenia in 1 patient, grade 2 dysphagia in 1 patient).

In contrast, like the patient described in our report, Kawamoto et al. observed severe acute enterocolitis in a patient treated with palliative RT (30 Gy/10 fractions for iliac and sacral metastases) concurrently with palbociclib‐fulvestrant.^13^ However, unlike the present case, the patient had previously experienced grade 1 diarrhoea on palbociclib. The patient recovered from the acute RT toxicity in 3 weeks with supportive care. We have briefly summarised the different studies describing the association of use of RT with CDK4/6 inhibitors in Table 1.

**Table 1:** Studies reporting use of CDK4/6 inhibitors along with radiotherapy in breast cancer.

Study	Patients	CDK4/6 inhibitors used	Radiation sites	Radiation dose	Radiation techniques	Toxicity profile
Hans et al.^12^	5	Palbociclib	Vertebra (2) Sacroiliac (1) Scapula‐humerus (1) Liver (1)	Bone metastasis (20 Gy/5 fractions) Liver‐60 Gy/10	Liver‐radiosurgery	Grade 3 neutropenia (2) Grade 3 anaemia (1) Grade 3 thrombocytopenia (2) Grade 2 mucositis (1) Grade 1 mucositis (1)
Kawamoto et al.^13^	1	Palbociclib	Pelvis	30 Gy/10	Conformal	Grade 1 diarrhoea (during RT) Grade 3 colitis (resolved with supportive care)
Meattini et al.^14^	5	Ribociclib	Femur (2) Hip (1) Lumbar vertebra (1) Cervical‐thoracic vertebra (1)	20 Gy/5 (4) 30 Gy/5 (1, femur neck)	3DCRT (4) VMAT (1)	Grade 3‐4 neutropenia (1) Grade 3‐4 diarrhoea/ vomiting (1, RT to hip)
Chowdhary et al.^15^	16	Palbociclib	Vertebra (9) Pelvic bone (2) Hip (2) Shoulder (1) Femur/ Knee (2) Ribs (1) Calvarium (1) Brain (4) Mediastinum (1)	Bone SBRT: 18 Gy/1; 30 Gy/3 Bone 3DCRT:30 Gy/10; 35 Gy/14; 37.5 Gy/15 Brain WBRT: 30 Gy/10: 35 Gy/14 Mediastinum: 36 Gy/18	Bones: 3DCRT‐15, SBRT‐2, IMRT‐1) Brain: WBRT‐3, Cavity SRS‐1 Mediastinum: IMRT	Grade 2 haematological toxicity (2) No grade 3‐4 haematological toxicity No grade 2‐4 cutaneous, neurological, gastrointestinal toxicity
Ippolito et al.^16^	16	Palbociclib (13) Ribociclib (3)	Thorax non‐spine (8) Pelvis non‐spine (6) Thoracic vertebra (4) Abdominal vertebra (4) Skin (1) IMN (1)	Median dose for palliative RT 30 Gy, range 8‐36 Gy) 5 patients with oligometastasis/ recurrence (sternum, spine, humerus, skin, IMN): median dose 50 Gy (range, 39.6‐60 Gy)	3DCRT (19) VMAT (3) IMRT (2)	Grade 3‐4 neutropenia (5) No grade 2‐4 anaemia, thrombocytopenia Grade 2 dermatitis (1, RT for skin) No other grade 2‐4 non‐haematological toxicity
Beddok et al.^17^	30	Palbociclib	LR (9) Bone (24) Brain (2)	LRRT‐50 Gy/25 (7) 50.4 Gy with SIB of 64.4 Gy in 28 (2) Metastatic sites 20 Gy/5 (13) 30 Gy/10 (10) 8 Gy/1 (3) SRS‐18 Gy/1 (1)	LRRT‐IMRT 3DCRT‐majority of RT to metastatic sites SRS for brain	Grade 2 or higher toxicities were seen in 13 patients (neutropenia in 9, dermatitis in 2, dysphagia in 1, pain in 1) Palbociclib was discontinued in 2 patients receiving LRRT
Current study	1	Palbociclib	Pelvis	30 Gy/10	2‐field opposing portals	Grade 3 colitis (resolved with supportive care)

CDK4/6 inhibitor: cyclin‐dependent kinase 4/6 inhibitor; RT: radiation; 3DCRT: 3‐dimensional conformal radiotherapy; VMAT: volumetric‐modulated arc therapy; SBRT: stereotactic body radiotherapy; WBRT: whole brain radiotherapy; SRS‐stereotactic radiosurgery; IMRT: intensity‐modulated radiation therapy; IMN: internal mammary node. LR: loco‐regional.

The baseline incidence of grade 3‐4 diarrhoea treated with CDK4/6 inhibitors alone was reported as < 5% of patients in a systematic review including 2007 patients.^23^ Therefore, the few reported instances of accelerated GI toxicity from pelvic RT‐palbociclib are likely a combined effect of the two anticancer treatments. Interestingly, a preclinical study had shown a protective effect of CDK4/6 inhibitors when delivered before a single fraction (SF) of RT, whereas fractionated RT led to an exacerbation of GI toxicity.^24^ A higher proportion of surviving crypts in the small intestine was associated with SF‐RT improving the integrity of the GI mucosal barrier, which was lost with multiple treatments likely resulting from impaired regeneration.

The use of palliative RT to the pelvis can lead to the development of acute colitis, although grade 3 toxicities are rarely encountered with the commonly used palliative dose fractionations. The concurrent use of palbociclib in our case should raise a strong index of suspicion for synergistic toxicity. Kawamoto et al. had witnessed GI toxicity with the use of conformal RT technique.^13^ It is not possible to conclude if conformal techniques would have had a clinical impact on reducing the likelihood of such toxicities. However, it will be reasonable to employ such techniques to reduce doses to bowel anticipating a dose‐dependent effect until stronger evidence is available. Similarly, there are no guidelines yet to suggest withholding the CDK4/6 inhibitors during RT can reduce toxicities. From the few instances of reported GI toxicity and with the introduction of more potent CDK4/6 inhibitors like abemaciclib (with higher events of GI toxicity), we empirically recommend the drugs should be avoided with RT.

Given the paucity of data and heterogeneity of treatments from the available literature, larger, possibly multi‐institutional studies are required to guide appropriate clinical practice. Here we postulate some strategies which can be adopted when pelvic RT is to be given to a patient taking a CDK4/6 inhibitor.

1. Consideration of stopping the CDK4/6 inhibitors 1 week before, during RT, and for 1 week minimally following the last day RT is delivered. Patients can continue endocrine therapy without interruption.
2. To plan for 5 fractions of RT (20 Gy/5 fractions) to minimise the treatment gap as opposed to relatively longer courses like 30 Gy/10 fractions.
3. Consideration of SBRT in appropriate patients (delivered in 5 or fewer fractions).
4. In patients with previous GI or haematological toxicities and GI comorbidities, careful monitoring of symptoms during RT and the following week. Consideration of extending the treatment break till all symptoms resolve (including minor effects).
5. Conformal techniques (intensity‐modulated radiotherapy/ volumetric‐modulated arc therapy) should be considered if that leads to a reduction of the bowel dose.

## Funding

No funding was involved with the current manuscript.

## Conflict of Interest

Archya Dasgupta: None; Arjun Sahgal: Advisor/consultant with Abbvie, Merck, Roche, Varian (Medical Advisory Group), Elekta (Gamma Knife Icon), BrainLAB, and VieCure (Medical Advisory Board), Board Member: International Stereotactic Radiosurgery Society (ISRS). Past educational seminars with Elekta AB, Accuray Inc., Varian (CNS Teaching Faculty), BrainLAB, Medtronic Kyphon. Research grant with Elekta AB. Travel accommodations/expenses by Elekta, Varian, BrainLAB. Elekta MR Linac Research Consortium, Elekta Spine, Oligometastases and Linac Based SRS Consortia; Ellen Warner: None; Gregory Czarnota: None.
